# Does green credit promote green sustainable development in regional economies?—Empirical evidence from 280 cities in China

**DOI:** 10.1371/journal.pone.0277569

**Published:** 2022-11-10

**Authors:** Jian Bao, Meiling He

**Affiliations:** 1 Institute of Chinese Financial Studies, Southwestern University of Finance and Economics, Chengdu, Sichuan, China; 2 School of Economics, Nanjing University of Posts and Telecommunications, Nanjing, Jiangsu, China; Hainan University, CHINA

## Abstract

**Background:**

China has been exploring a sustainable development path that harmonizes economic growth and environmental protection, targeting to build a beautiful China. The role of green finance in adjusting the misallocation of financial resources and leading the green sustainable development of the real economy is receiving increasingly more attention. Currently, green credit accounts for more than 90% of the total green finance funding in China and constitutes the most significant component of the green finance matrix. Whether green credit effectively promotes the green and sustainable development of the regional economy largely determines the success of China’s economic green transformation.

**Objective:**

Existing studies of green credit mainly focus on its influences on financing, investment, and emission reduction of environmental pollution industries or companies. Extending the literature by exploring whether green credit is effective in promoting green sustainable development and what impact green credit exerts on the upstream (energy inputs), midstream (technological innovation), and downstream (pollution outputs) stages of the green sustainable development value chain, is the leading research objective of this paper.

**Methods:**

This paper discusses the impact of green credit on green sustainable development based on city panel data from 2012 to 2019. The level of green sustainable development is calculated by the GML index based on SBM directional distance function. The city-level green credit scale is calculated from the green credit issued by banks, weighted by the density of bank branches in a city. Synthetic control methods are employed in the robustness analysis to reduce the impact of endogeneity issues.

**Results and conclusion:**

The results of this paper indicate that green credit can promote green sustainable development and the impact gradually strengthens over time as the incremental implementations of complementary policies with substantial constraints and incentives, through which pollution control and economic growth achieve a "win-win" situation. Furthermore, the results indicate that green credit reduces the overall amount of energy inputs while optimizing the energy input structure. However, green credit does not boost the green technological level and even crowds out high technical value green innovations. Besides, the pollution reduction effects of green credit are associated with the strength of green credit constraints and the importance of pollution industries in the local economy, which means green credit performs better pollution reduction effects in regions with relatively strong green credit binding effects or in regions where pollution industries are not local economic pillars. The empirical results are further validated through robustness tests, including changing scope and measurement variables and applying the synthetic control method.

**Limitations:**

Although this paper provides valuable contributions to the research area of green credit and green sustainable development, specific limitations exist in the current study. Firstly, as the official information disclosure of green credit in China is not sufficient, existing studies, including ours, could only use estimation methods through different perspectives to measure green credit, which is overall logical and reasonable but may lose some accuracy. Secondly, since there might be a certain degree of lag in the effect of green credit on the economy, the dynamic impact and long-term effects of green credit deserve further study. Thirdly, considering the characteristics of the Chinese administrative systems, introducing the behavior of local governments and local officials into the analysis of green credit and green sustainable development could be valuable.

## Introduction

At different stages of economic and financial development in China, exploring a balanced path that harmonizes economic growth and environmental protection has always received considerable attention in the literature. Since the Reform and Opening-up policy, China has embarked on a high-speed development road with industrialization and urbanization. While the economic development gains expected growth rate, the ecological environment has deteriorated rapidly, and the environmental carrying capacity is gradually closing to its limit in recent years. The presence of the Environmental Kuznets Curve [1] calls for the preventive green transformation of China’s economic development model, before reaching the inflection point of economic growth. In this context, it is vital for China’s economy to shift to a more scientific development mode with green sustainability as its core. In the green sustainable transformation of the economy, one of the most significant issues that need to cope with carefully is to vigorously practice environmental protection without sacrificing economic growth.

Traditional environmental regulatory measures are either based on incentives such as government environmental subsidies or punishments such as environmental penalties and pollution fees. On the one hand, government environmental subsidies cannot benefit all relevant enterprises, and their effectiveness in constraining the operation behavior of high-pollution and high energy-consuming enterprises is also limited. On the other hand, the Pollution Haven Hypothesis shows that the relative stringencies of regional environmental regulations may only lead to the re-locations of polluting enterprises and the cross-regional flow of financial resources channeled to polluting industries, failing to address the root causes of ecological problems [1, 2]. Therefore, the effectiveness of traditional environmental regulations with purely punitive or incentive effects is not satisfactory.

The establishment and flourishing of a sound green sustainable economic development model could hardly realize without an effective financial system. Review the rapid economic development in the past decades, substantial financial resources have flowed into industrial sectors with intensive energy consumption and high pollution discharge, which exacerbates environmental pollution along with overcapacity. With the traditional financial system seemingly failing to balance economic development and environmental protection, green finance is increasingly expected to effectively promote China’s green sustainable economic transformation. Green finance aims to organize and direct financial resources to promote sustainable economic development and conservation of natural ecology through green credit, green bonds, green insurance, green equity, and other financial instruments.

In China’s bank-dominated financial system, green credit is undoubtedly the most critical component of green finance. Compared with other environmental regulation measures, green credit is a "carrot and stick" type measure with positive incentives and negative penalties. Specifically, for enterprises committed to environmental protection, energy conservation, and emissions reduction, green credit facilitates their access to financial resources and reduces corporate financing costs; for enterprises characterized by high energy consumption and pollution discharge, green credit imposes punitive measures by raising loan thresholds and increasing loan interest rates. In addition, compared to administrative penalties and shutdown orders, green credit is more flexible and market-oriented through the regulation of loan availability, loan size, loan maturity, and loan rates.

Currently, China leads the world on the scale of green credit. According to data released by the People’s Bank of China, as of end-2021, green loans in RMB and foreign currencies posted an outstanding amount of RMB 15.9 trillion, rising 33 percent year on year, an acceleration of 12.7 percentage points from the end-2020 [3]. While green credit has gained vigorous development in recent years, research in this area is relatively insufficient in China. In terms of research content, most relevant studies mainly focus on the "pollution reduction effect" (whether green credit can reduce pollution emissions), the "growth effect" (whether green credit can influence economic growth), and the "Porter effect" (whether green credit can stimulate technological innovation) at the enterprise level.

For the pollution reduction effect, studies have found that financial development is one of the main driving forces leading to environmental degradation [4], which is particularly typical in the process of China’s heavy industrialization. On the other hand, as a financial creation, the development of green credit has been verified to be beneficial for the ecological environment and pollution control [5, 6]. Zhang et al. [7] indicated that as part of the green finance index, green credit can effectively curb carbon emissions. Gao [8] pointed out that green credit policy improves the environmental protection behaviors of high-polluting enterprises.

Regarding the growth effect, green credit policies limit the financial resource availability and production expansion of pollution-intensive enterprises while restraining the growth of polluting industries [9, 10]. On the contrary, green credit enhances the value of new energy companies, which is characterized by environmentally friendly attributes by easing financing constraints and strengthening external oversight [11]. In addition, the resource allocation, credit catalysis, and policy guidance effects of green credit, positively drive the rationalization, advancement, and greening of industrial structure [12]. However, The strategic responses of enterprises in facing financial constraints that the green credit policy imposes, to some extent, affect the positive effects expected from green credit [13]. Besides, alternative sources of financing that enterprises can exploit, such as commercial credit, may significantly undermine the desired resource allocation effectiveness of the green credit policy [14].

According to Porter’s hypothesis, there are certain intrinsic connections between environment, productivity, innovation, and competitive advantage. If an environmental protection initiative is formulated effectively, it could motivate enterprises to invest in innovation and increase productivity, which could offset environmental compliance costs. Through this path, the environmental protection initiative contributes to a win-win scenario where the goals of environmental protection and economic development are both achieved [15]. Whether Porter’s hypothesis can be verified in the practice of green finance in China has gained considerable research attention. Some studies proposed that green credit fails to contribute to, and even negatively influence, green innovation due to compliance costs and credit constraints caused by green credit policies [16, 17]. In comparison, other studies suggested that green credit stimulates enterprise innovation, especially when the sunk costs, noncompliance costs, and degree of market competition are unfavorable [18]. The supervisory function of green credit raises shareholders’ attention to the transformation of corporate production methods toward energy conservation, emission reduction, and low-carbon, which imposes high expectations on management’s environmental governance decisions to promote green innovation investments and seek green development opportunities [19].

The ultimate intention of the government’s implementation of green credit policies is not to suppress manufacturing production, delay technological innovation and stall business development, but to integrate ecological and environmental factors into the operations of each relevant enterprise, encourage the transformation and upgrading of pollution and energy-intensive business model, and collectively realize the green sustainable development. Considering the large scale of green credit issuance in China, the question of whether green credit is functional in promoting green sustainable development with an effectively coordinated relationship between economic growth and environmental protection needs to be answered urgently. However, among the reviewed literature, only a few studies have empirically investigated the relationship between green credit and green sustainable development [20]. To expand current research boundaries and better answer the targeted question, the measurement of regional green credit deserves further exploration, and the role of green credit in the upstream (energy inputs), midstream (technological innovation), and downstream (pollution outputs) stages along the green sustainable development value chain merit in-depth dissection and analysis.

Although there are various studies on the effects of green credit in China, in terms of research methods, more studies employ the difference-in-difference (DID) technique to compare the "pollution reduction effect" or "Porter effect" on polluting enterprises or industries before and after the implementation of green credit policies to determine the effectiveness of green credit. In terms of the research conclusions, findings are inconsistent or even contradictory. The diverging conclusions on the effects of China’s green credit are mainly attributed to the following reasons. First, the selected green credit policies used for quasi-natural experiments are not identical. Some studies select the *Opinions on Implementing Environmental Protection Policies and Regulations to Prevent Credit Risks* promulgated in 2007 [16] as the landmark of China’s implementation of green credit policy, while others select the *Green Credit Guidelines* promulgated in 2012 [21]. Second, the treatment groups and control groups were established in different ways. Some studies establish treatment groups according to features of high pollution, high energy consumption, and overcapacity industries or based on the *List of Classified Management of Environmental Protection Industry of Listed Companies* promulgated by the Ministry of Environmental Protection in 2008 [22], while others establish treatment groups through specific measurement methods of sample pollution emissions data [18]. Third, the elements studied differ in their measurement, representation, and conceptual scope. To illustrate, some studies proposed that green credit supports the green economic behavior of enterprises, so using green patent applications to measure green innovation is appropriate [23]. As a comparison, some studies argued that enterprises subject to green credit constraints seek to innovate in all possible dimensions, so the level of innovation is more reasonable to be measured by the quantity of all patents registered [16]. Besides, some other studies demonstrated Porter’s hypothesis by observing the impact of environmentally induced R&D on firm-level green productivity [24]. Even though the DID model has been favored in studying the policy impact of green credit on high-pollution enterprises and industries, the model has limitation in assessing the overall effect of green credit on the economy. For example, green credit policies might indeed sustain the green transformation and upgrading of polluting enterprises, but they could also crowd out the loan resources of other industries simultaneously, thus the overall impact on green economic development remains ambiguous.

Exploring whether green credit is effective in promoting green sustainable development and what impact green credit exerts on the upstream (energy inputs), midstream (technological innovation), and downstream (pollution outputs) stages along the green sustainable development value chain, are the leading research objectives of this paper. Compared with existing studies, this paper presents the following contributions. First, this paper expands the research boundaries with a fresh perspective on the relationship between green credit and regional green economic development, which complements existing views on the impact of green credit policy on polluting enterprises or industries. The green total factor productivity (GTFP) is employed in this paper as the proxy variable for the degree of green sustainable development at the city level, serving as a more comprehensive indicator, taking into account both the desired output, represented by GDP growth, as well as the undesired output, represented by pollution emissions, while considering energy consumption. Second, the potential impacts of green credit exert on the upstream (energy inputs), midstream (technological innovation), and downstream (pollution outputs) stages along the green sustainable development value chain are further examined in this paper. The findings related to each stage provide insightful references for policymakers to develop targeted improvements to the green finance institutional system. Third, this paper extends the study of the impact of green credit from enterprises to regions, which is helpful in systematically understanding the impact of green credit on the economy. Since many studies have examined the role of green credit from the perspective of listed companies, few studies have discussed the effectiveness of green credit at the regional level. Fourth, this paper enriches the research methods with a novel approach to measuring regional green credit. The green credit policies issued in 2007 or 2012 have been extensively used by existing literature as breakpoints to identify the effect of green credit. But the policy shocks have passed more than ten years, and whether they are representative enough to explain the effect of green credit in recent years is questionable. In this paper, green credit data of each bank from 2012 to 2019 are manually collected, and the scale of green credit at the city level is calculated based on the weight of the branch density of each bank in each city, which lays the foundation for re-identifying the role of green credit at the city level. Fifth, this paper applies the synthetic control method to reduce the endogenous effect by taking green finance reform and innovation pilot zones implemented since 2017 as policy shocks. This method examines the impact of green credit at the regional level, without the artificial division between high-polluting and low-polluting sectors as in the DID method.

## Theoretical analysis and hypotheses

The issuance of green credit is a way for commercial banks to actively meet financial regulation and monetary policy and assume environmental and social responsibility while pursuing lending profit [25]. Creditors’ considerations in issuing green credit include the repayment capability of the borrower and the borrower’s environmental performance as well [26]. With the reinforcement of green credit policies and the enhancement of ecological awareness of financial institutions, financial resources are gradually withdrawn from high energy-consuming and high-polluting sectors and redirected to energy conservation and environment preservation sectors, through which the financial system promotes the green transformation of the economy from brown to green through the optimized allocation of financial resources. With the direct effects of green credit, the investment and financing of polluting enterprises are constrained, the expansion rate of polluting projects decreases, the financial performance deteriorates, and the market competitiveness declines. While for the environmentally friendly enterprises that are encouraged by green credit policies, their credit availability is enhanced, operational productivity is promoted, and market position is strengthened. With the indirect effects of green credit, the warning signal to high-pollution enterprises and bonus signal to environmentally friendly enterprises prompt the overall corporate sector to establish environmental awareness, care about energy efficiency and emission control, and actively assume environmental responsibilities. In summary, green credit is theoretically conducive to ecological and environmental protection.

Can green credit promote economic development while protecting the ecological environment? This question is of particular importance for China that under significant environmental pressure and possesses the world’s leading green credit scale. Traditional environmental regulations have made relevant contributions to protecting the ecological environment. But the unintended effects incurred, such as transboundary pollution or downstream pollution, as well as the shutdown or evasive re-locations of enterprises [27–30], weaken the effectiveness of environmental regulation. It is because the traditional environmental regulations featuring punitive measures are more active in raising the operational costs of polluters, but lack substantial support for their green transformation. Compared with traditional environmental regulations, differentiated green credit policies reduce policy-encouraged enterprises’ environmental compliance costs through improved financing conditions and lowered financing cost, which comprehensively ensures their profit margins and market competitiveness are not significantly affected by environmental governance. In addition, administrative penalties and emission fees only push polluting enterprises to meet specific standards, while green credit can influence all enterprises that need bank financing. To continuously obtain financing support from banks, enterprises should not only compy with environmental standards in the short term but also make continuous efforts to transform towards a green sustainable direction in the long run.

Although China’s green credit policies have been implemented and China has been leading the world in the scale of green credit, the current green credit practices in China are still far from perfect. First, the strategic responses of enterprises may weaken the effectiveness of green credit. Some enterprises may greenwash business activities and make misleading environmental disclosures to access green financing [31]. Second, how well the execution of green credit policies by banks could affect the effectiveness of green credit. China’s green credit system lacks explicit and uniform standards. The stringency of green credit approval and post-loan supervision that ensure loans are used for green development may vary among banks [32]. Under the risk capital constraint of banks, excessive issuance of green credit may crowd out other credit products, leading to a credit structure that deviates from the optimal and causes overcapacity in the green industry. Considering the potential problems discussed above, it is questionable whether green credit can have a positive impact on green sustainable development. Consequently, the following competing hypotheses are proposed:

Hypothesis 1a: Green credit effectively promotes green sustainable development.

Hypothesis 1b: Green credit does not promote green sustainable development.

In the past, environmental regulations have generally focused on the control of pollutant emissions, yet through which it is difficult to essentially shift the growth model from "pollute first, treat later" to green sustainable development. While implementing green credit policies, through the process of credit approval and post-loan supervision, banks assess the repayment capability and environmental performance of enterprises through the comprehensive analysis of their value chain and business cycle. As such, what impact green credit exerts on the upstream (energy inputs), midstream (technological innovation), and downstream (pollution outputs) stages along the green sustainable development value chain, is worth further study.

First, the role of green credit in energy inputs at the upstream stage of the green sustainable development value chain is analyzed. In regions with strictly enforced green credit policies and a relatively higher proportion of green credit, high-energy-consuming projects are more difficult to access loans and phased out at an accelerated rate, while clean energy projects bloom with incremental financing support, driving the economy to develop sustainably [33]. In addition, green credit leads fund flow into energy conservation industries, improving energy consumption structure and efficiency [34]. Besides, since bank credit is the most critical financing tool for enterprises in China, commercial banks’ increasing attention to corporate energy performance can be instructive to other investors’ financing decisions, forcing enterprises to take more practical actions to improve energy performance. Regarding this, the following competing hypotheses are proposed:

Hypothesis 2a: Green credit promotes green sustainable development at the upstream stage by optimizing energy inputs.

Hypothesis 2b: Green credit has no impact on energy inputs at the upstream stage of green sustainable development.

Second, the role of green credit in technological innovation at the midstream stage of the green sustainable development value chain is analyzed. Referring to the results from existing studies on environmental regulation, some studies propose that under strict environmental regulation, enterprises inevitably incur increased compliance costs to meet environmental standards, such as installing required controlling equipment and upgrading production processes. These additional costs lead to crowding-out effects on enterprises’ capability and willingness for other operational activities, including technological innovation, capital investment, and foreign trade [35, 36]. Other studies argued that strict environmental regulations lead to higher cost burdens related to environmental violations and force enterprises to attach more importance to technological innovation [37]. The core difference between these two views is whether innovative activities are crowded out or prioritized under strict environmental regulations. In the game of environmental protection between enterprises and the government, the government does not expect enterprises to close down or relocate because of environmental regulations. The essential purpose of the regulations is to guide enterprises to transform and shift to the green sustainable development path. Transformation and redirection require innovation as driving forces, while research and development need funding support. Green credit, as a market-based environmental regulation, can reduce enterprises’ barriers to participation in green research and development by improving the availability and accessibility of credit funds. Regarding this, the following competing hypotheses are proposed:

Hypothesis 3a: Green credit promotes green sustainable development at the midstream stage by fostering green innovation.

Hypothesis 3b: Green credit has no impact on green innovation at the midstream stage of green sustainable development.

Finally, the role of green credit in pollution output at the downstream stage of the green sustainable development value chain is analyzed. Pollution control in a region depends on two factors: first, the reduction of pollutants at the enterprise level; second, the shrinking of the market share of high-pollution industries at the industrial structure level. At the enterprise level, green credit supports enterprises’ capital investments and upgradation related to pollution reduction while restraining the financing of pollution-intensive projects, achieving the pollution reduction goal [10]. In addition, commercial banks’ gradually enhanced awareness of ESG investment sends clear signals to the credit market that enterprises must make continuous efforts to improve their environmental performance to maintain their credit lines. Under the pressure of external financing, polluters need to take the initiative to be environmentally responsible and invest in pollution reduction facilities for long-term development. At the industrial structure level, as credit resources keep flowing out from high-pollution industries and into green credit-supported sectors such as clean energy, new energy vehicle, and green service, industrial structure achieved green optimization [38]. But in practice, the actual effects of green credit could be disturbed by the behavior of government, enterprises, and commercial banks [39]. Regarding this, the following competing hypotheses are proposed:

Hypothesis 4a: Green credit promotes green sustainable development at the downstream stage by controlling pollution outputs.

Hypothesis 4b: Green credit has no impact on the downstream stage of green sustainable development.

## Research design

### Sample selection and data sources

The city-level green credit indexes are calculated from the green credit balances of 3 policy banks and 67 commercial banks. Among them, the data of 21 leading banks (3 policy banks and 18 commercial banks) are from the CSMAR database, and the data of the remaining 49 banks are manually collected from financial reports, social responsibility reports, sustainability reports, official websites or interview. Data related to energy and emissions are from the *China Statistical Yearbook on Environment* and *China Energy Statistical Yearbook*. Bank branch data are from financial license disclosures of the China Banking and Insurance Regulatory Commission. Other regional data are from the *China Statistical Yearbook* and the CSMAR database.

This paper uses 280 cities in China from 2012 to 2019 as samples to analyze whether green credit promotes the green sustainable development of the economy at the city level. The year 2012 is chosen as the starting point because this was the year that the *Green Credit Guidelines* were issued, which specifies the principles for granting green credit and has been widely documented as the landmark of the green credit policy’s formal implementation in China.

### Index selection and construction

#### Dependent variable: Green sustainable growth

The construction of green sustainable growth indexes requires simultaneous consideration of environmental protection and economic development. Green Total Factor Productivity (GTFP) is employed in this paper to synthesize the targets. While the general total factor productivity (TFP) only considers the maximizing outputs under the condition of given factor inputs. GTFP further considers the realization of output maximization under the conditions of low energy consumption and low pollution, which effectively introduce environmental factors into the growth model.

The Data Envelopment Analysis (DEA) is an important method for GTFP by measuring the relative effectiveness among multiple decision-making units (DMU) based on input and output data. It possesses the advantages of not requiring a specific form of production function and the capability of directly handling multiple inputs and outputs. When the data of the DMU evaluated are panel data containing various time points and observations, the measurement of productivity changes is the Malmquist index analysis. However, there are two issues with the Malmquist index. First, if there is non-zero slack in the inputs or outputs, the radial directional distance functions will underestimate the technology inefficiency due to failing to capture the slacks. Second, the Malmquist index does not consider the effect of undesirable outputs, which potentially leads to overestimates of efficiency. To effectively estimate GTFP, this paper calculates the Global Malmquist-Luenberger (GML) productivity index based on the non-radial and non-angular slacks-based measure (SBM) directional distance function, forming the SBM-GML model. Compared with the general Malmquist index, the SBM-GML model has the following advantages. First, SBM-GML properly considers the effect of undesirable outputs resulting from environmental degradation, which enhances the comprehensiveness of productivity evaluation under the DEA framework. Second, the non-radial and non-angular SBM can mitigate the estimation bias of the radial model when slack variables exist [40–42].

In the SBM-GML model, M kinds of desirable outputs $y=(y_{1},\cdots ,y_{m},\cdots ,y_{M})\in R_{M}^{+}$ as well as I kinds of undesirable outputs $b=(b_{1},\cdots ,b_{i},\cdots ,b_{I})\in R_{I}^{+}$ are produced by each of the K DMUs at time t on the condition of N kinds of factor inputs $x=(x_{1},\cdots ,x_{n},\cdots ,x_{N})\in R_{N}^{+}$. The SBM directional distance function, which takes into account the pollutant emissions as undesirable outputs, can be described as:

$$\begin{array}{l} \;{\vec{S}}_{V}^{t}(x^{t,k^{\prime }},y^{t,k^{\prime }},b^{t,k^{\prime }},g^{x},g^{y},g^{b})\\ ={max}_{s^{x},s^{y},s^{b}}\frac{1}{2}\left\{\frac{1}{N}\sum_{n=1}^{N}\frac{s_{n}^{x}}{g_{n}^{x}}+\frac{1}{M+I}(\sum_{m=1}^{M}\frac{s_{m}^{y}}{g_{m}^{y}}+\sum_{i=1}^{I}\frac{s_{i}^{b}}{g_{i}^{b}})\right\} \end{array}$$ (1)

$$s.t\cdot \left\{ \begin{array}{l} \sum_{k=1}^{K}\gamma_{k}^{t}x_{kn}^{t}+s_{n}^{x}=x_{k^{\prime }n}^{t},\forall n;\\ \sum_{k=1}^{K}\gamma_{k}^{t}y_{km}^{t}-s_{m}^{y}=y_{k^{\prime }m}^{t},\forall m;\\ \sum_{k=1}^{K}\gamma_{k}^{t}b_{ki}^{t}+s_{i}^{b}=b_{k^{\prime }i}^{t},\forall i\\ \sum_{k=1}^{K}\gamma_{k}^{t}=1,\gamma_{k}^{t}⩾0,\forall k;\\ s_{n}^{x}⩾0,\forall n;\\ s_{m}^{y}⩾0,\forall m;\\ s_{i}^{b}⩾0,\forall i \end{array}\right.$$

Where $\left(x^{t,k^{\prime }},y^{t,k^{\prime }},b^{t,k^{\prime }}\right)$ is the vector that includes elements of factor inputs, desirable outputs, and undesirable outputs, respectively; (g^x^,g^y^,g^b^) is the vector that indicates the contraction of factor inputs, the expansion of desirable outputs, and the contraction of undesirable outputs, respectively; $\left(s_{n}^{x}{,s}_{m}^{y},s_{i}^{b}\right)$ is the slack vector of factor inputs, desirable outputs, and undesirable outputs; and ${\vec{S}}_{V}^{t}$ denotes the SBM directional distance function under the assumption of variable returns to scale of production [42].

In our calculation of GTFP, the city is the DMU, and the factor inputs include labor, capital, and energy. The labor factor is evaluated by the number of employees in urban areas. The capital factor is calculated based on the Perpetual Inventory Method, with the net fixed asset investment in 2000 as the base period capital stock and the 9.6% capital depreciation rate, consistent with the estimation of Zhang et al. [43].


$$K_{it}=K_{it-1}\times (1-\sigma )+I_{it}/P_{it}$$ (2)

Where K_it_ indicates the capital stock of region i in period t, K_it−1_ indicates the capital stock of region i in period t-1, σ is the capital depreciation rate, I_it_ indicates the amount of fixed asset investment of region i in period t, P_it_ is the fixed asset investment price index of region i in period t. Fixed asset investment data and fixed asset investment price index are from the CSMAR database. We use the provincial fixed asset investment price index to proxy the city-level fixed asset investment price index due to the availability of data.

The energy factor is denoted by electricity consumption, which overcomes the lack of city-level fossil energy consumption data. The desirable output variable is the actual GDP, which is calculated by deflating the nominal GDP of each city to 2000 using the GDP deflator. The undesirable output is environmental pollutants, including industrial emissions of sulfur dioxide, soot, and wastewater. Relevant data are obtained from the *city statistical yearbooks*. The GTFP is calculated using MaxDEA software.

#### Independent variable: Green credit

The bank-level green credit balances are manually collected from financial reports, social responsibility reports, sustainability reports, and official websites. According to financial institutions’ operating logic of business branches, a bank establishes more branches in regions with high business demand and fewer branches in regions with low business demand, dynamically forming a relatively balanced business volume among each branch. Green credit, in common with other banking products, is operated by each branch. This means the scale of green credit issuance should have a positive correlation with the density of bank branches. In addition, the implementation of green credit in China carries a degree of political requirements. Most Chinese banks, as state-owned enterprises, need to respond strictly to governments’ and banking regulators’ requirements to grow green credit business, such as the requirement “the growth rate of green loan shall not be lower than the average growth rate of loans” by banking regulators. While receiving these requirements, banks will routinely break down and distribute tasks to each branch, further enhancing the positive correlation between the green credit scale and the density of bank branches. Therefore, with reference to Jiang et al. [44], we take the number of branches of each bank in a city as a proportion of the total number of branches of the bank as weights to calculate the green credit contribution of the bank to various cities. Then accumulate each bank’s city-level green credit contribution to obtain the total green credit scale of the city.


$${gcredit}_{ijt}={gcredit}_{it}*\frac{num_{ijt}}{num_{it}}$$ (3)

$${gcredit}_{jt}=\frac{\sum_{i}{gcredit}_{ijt}}{{tcredit}_{jt}}$$ (4)

In Eq (3), *i*, *j* and *t* represent the commercial bank, the city, and the year, respectively. Therefore, *gcredit*_*it*_ represents the scale of green credit of year t issued by commercial bank *i*; *num*_*ijt*_ represents the number of branches of commercial bank *i* in city *j* in year *t*; *num*_*it*_ represents the total number of branches owned across the country by commercial bank *i* in year *t*. Further, $\frac{num_{ijt}}{num_{jt}}$ is the proportion of branches bank *i* operates in city *j*, and this proportion is used as the weight to distribute a bank’s green credit scale to each city. Consequently, *gcredit*_*ijt*_ is the green credit scale issued by bank i in city j in year t. At the city level, Eq (4) sums up *gcredit*_*ijt*_ in the city dimension and obtains the city-level green credit scale. Finally, the ratio of the city-level green credit balance (∑_*i*_*gcredit*_*ijt*_) to the year-end RMB loan balance of financial institutions (*tcredit*_*jt*_) in each city is used as the green credit variable (*gcredit*) in region *j*.

#### Control variables

In this paper, we control as many variables as possible at the regional level to reduce the influence of confounding factors, which include: (1) GDP per capita; (2) the share of secondary industry in GDP; (3) the share of tertiary industry in GDP; (4) the degree of financial development, measured by the ratio of the RMB loan balance to GDP; (5) the degree of government intervention, measured as the difference between fiscal revenue and expenditure as a percentage of fiscal expenditure; (6) education level, measured by the proportion of education spending in fiscal expenditure; (7) technological level, measured by the proportion of technology spending in fiscal expenditure; (8) openness level, measured by the amount of actual foreign investment used in each year as a percentage of GDP; (9) domestic trade, measured by total retail sales of consumer goods as a percentage of GDP; (10) infrastructure, measured by urban road area per capita and the degree of internet development; and (11) the stringency of local government environmental regulation, measured by text analysis on the manually collected city governments’ work reports, to identify the word density calculated by the ratio of ecological environment keywords, such as "PM10", "low carbon", "emission reduction", "green" and "environmental protection", to the total words of the report [45]. S1 Table summarizes the units, data sources, measurements, and time periods of the variables.

### Econometric model

With the panel fixed-effect model, which simultaneously controls the city fixed effect and the year fixed effect, this paper discusses the impact of green credit on green sustainable development based on city panel data from 2012 to 2019. The econometric model is defined as:

$$gtfp_{jt}=\alpha +\beta \times gcredit_{jt}+\sum \gamma \times control_{jt}+u_{j}+u_{t}+\varepsilon_{jt}$$ (5)

Where j represents city and t represents year, a positive *β* coefficient indicates a positive relationship between green credit and GTFP. City-level clustering robust standard error is examined.

### Descriptive statistics

Table 1 summarizes the descriptive statistical results of the variables.

**Table 1:** Descriptive statistical results.

Variable	Unit	Observations	Mean	Median	Standard deviation	Minimum	Maximum
**GTFP**	N.A.	2155	1.01	1	0.08	0.42	1.26
**Green credit**	1	2155	0.15	0.13	0.1	0	0.73
**GDP per capita**	Million	2155	10.74	10.66	0.67	8.07	13.21
**Share of secondary industry in GDP**	1	2155	0.46	0.47	0.1	0.12	0.81
**Share of tertiary industry in GDP**	1	2155	0.42	0.41	0.1	0.15	0.84
**Degree of financial development**	1	2155	1	0.83	0.62	0.12	9.62
**Degree of government intervention**	1	2155	-0.53	-0.57	0.22	-0.93	0.54
**Education level**	1	2155	0.18	0.18	0.04	0.01	0.36
**Technological level**	1	2155	0.02	0.01	0.02	0	0.21
**Openness level**	1	2155	0	0	0	0	0.02
**Domestic trade**	1	2155	0.39	0.39	0.11	0	1.01
**Urban road area per capita**	M^2^	2155	16.68	15.42	4.22	4.08	26.2
**Degree of internet development**	1	2155	0.24	0.19	0.18	0.01	1.95
**stringency of local government environmental regulation**	1	2155	0.01	0.01	0	0	0.02

Note: The use of 1 as a unit is because the variable is a ratio value and the numerator and denominator involved in the calculation have the same unit.

## Empirical results

### Basic regression results

Table 2 shows the regression results of GTFP on green credit at the city level. Column (1) is the result when control variables are not included, and the coefficient of green credit is significantly positive at the 1% level. Column (2) is the result of controlling for the city fixed effect and year fixed effect, and the coefficient of green credit is significantly positive at the 5% level. The smaller coefficient of green credit in column (2) compared with column (1), indicates that a portion of the influence of green credit on GTFP is absorbed by fixed effects. Based on column (2), the complete set of control variables is further controlled in column (3). The coefficient of green credit remains significantly positive at the 5% level, and the coefficient value is close to that in column (2).

**Table 2:** Basic regression results for the impact of green credit on GTFP.

	(1)	(2)	(3)	(4)	(5)
	*gtfp*	*gtfp*	*gtfp*	*gtfp*	*gtfp*
*gcredit*	0.150***	0.057***	0.059***		
	(7.69)	(2.81)	(2.69)		
gcredit*d1				-0.093*	-0.096*
				(-1.76)	(-1.70)
gcredit*d2				0.050**	0.052**
				(2.12)	(2.20)
gcredit*d3				0.063*	0.069*
				(1.84)	(1.85)
Control variable	No	No	Yes	No	Yes
Urban fixed effect	No	Yes	Yes	Yes	Yes
Year fixed effect	No	Yes	Yes	Yes	Yes
*N*	2155	2155	2155	2155	2155
Adj.R^2^	0.034	0.182	0.184	0.186	0.187

Note:

***, **, and * mean significant at the 1%, 5%, and 10% levels, respectively; the values in parentheses are the t values adjusted by the clustering standard error; Adj.R^2^ represents adjusted R^2^.

To further explore whether the effect of green credit upon GTFP varies over time, the observation years are divided into three segments by the crucial milestones during the development of green credit in China. The first segment is 2012–2015, represented by the dummy variable *d1*. During this period, although the *Green Credit Guidelines* released in 2012 aroused banks’ attention to the green credit business, banks did not vigorously implement it due to a lack of substantive constraints and incentives [46]. The second segment is 2016–2017, represented by the dummy variable *d2*. In 2016, the People’s Bank of China, the Ministry of Finance, as well as other relevant ministries jointly formulated *the Guiding Opinions on Building a Green Financial System*, which marked the consensus from China’s highest political and strategic level to determine to fully support and promote green investment and financing in China and accelerate the transformation of the economy to green sustainable development. Since the third quarter of 2017, banks’ green credit performance has been formally included in the Macro Prudential Assessment (MPA) by the People’s Bank of China, which imposed strong regulatory constraints on banks’ development of green credit business as the result of the MPA assessment will directly affect the legal reserve rate of financial institutions. The third segment is 2018–2019, represented by the dummy variable *d3*. In 2018, high-quality green loans were accepted as qualified collateral for the innovative monetary policy tool medium-term lending facility (MLF) by the People’s Bank of China, providing banks substantial incentives to expand green credit.

Columns (4) and (5) present the results of the effect of green credit on GTFP over time, with column (4) controlling for the city fixed effect and the year fixed effect only, and column (5) further controlling the control variables in our econometric model. The magnitude and significance of the regression coefficients in Columns (4) and (5) are relatively consistent. The coefficient of gcredit*d1 is negative at the 10% significance level, indicating green credit was not conducive to GTFP advancement in 2012–2015. It is potential because at the early stage after the release of the green credit policy, banks’ attention to green credit is insufficient, and policy compliance is inadequate. During this transition period, environmentally friendly enterprises have not yet benefited significantly from green credit, while polluting enterprises rush to accelerate financing and development activities for fear of increasingly stringent green policy enforcement. The coefficients of gcredit*d2 and gcredit*d3 are both positive, confirming green credit boosted GTFP after 2015. Moreover, the coefficient of gcredit*d3 is more significant than that of gcredit*d2, indicating the positive impact of green credit on GTFP has increased over time, in correspondence with the incremental implementations of more complementary policies with substantial constraints and incentives effects. To summarize, green credit can promote green sustainable development and with the gradual improvement of the green credit policy system in recent years, the active impact of green credit on green sustainable development has become increasingly noticeable.

### Test of the full-value-chain role of green credit

The basic regression results indicate that green credit can promote green sustainable development. But whether green credit could break through the limitations of traditional regulatory measures that only act on the stage of pollution control, and effectively facilitate the transformation of the unsustainable "polluting first & treating later" economic development model, needs to be further explored. Regarding this, the impact of green credit on energy inputs, technological innovation, and pollution outputs is further studied to evaluate the role of green credit in the upstream, midstream, and downstream stages of the green sustainable development value chain.

#### Energy inputs and green credit

Energy supplies the raw power for economic development but is also an important source of pollution. The volume and structure of energy consumption affect economic development and ecological environment simultaneously. Table 3 shows the regression results of energy inputs on green credit. Since the city-level energy consumption data are available only for electricity and the information is limited, the provincial energy consumption data are selected as the explained variable, and the data are from the *China Energy Statistical Yearbook*. Column (1) shows the results of the impact of green credit on energy consumption from the perspective of total consumption. The explained variable is total energy consumption, calculated by first converting the eight major types of energy consumption (coal, electricity, crude oil, etc.) into million tons of standard coal equivalence and then normalizing it by the regional GDP. Columns (2) to (4) provide results on the role of green credit on energy consumption at the structural level. The explained variables are the proportion of consumptions of various types of energy, which are calculated by first converting the consumptions observed into million tons of standard coal equivalence and then normalizing them by the total energy consumption.

**Table 3:** Impact of green credit on energy inputs.

	(1)	(2)	(3)	(4)
	Total energy consumption	Proportion of clean energy	Proportion of coal	Proportion of petroleum
*gcredit*	-0.420*	0.085	-0.015**	-0.041**
	(-1.92)	(1.48)	(-2.14)	(-2.58)
Control variable	Yes	Yes	Yes	Yes
Urban fixed effect	Yes	Yes	Yes	Yes
Year fixed effect	Yes	Yes	Yes	Yes
*N*	1769	1885	1889	1773
Adj.R^2^	0.375	0.263	0.763	0.228

Note:

***, **, and * mean significant at the 1%, 5%, and 10% levels, respectively; the values in parentheses are the t values adjusted by the clustering standard error; Adj.R^2^ represents adjusted R^2^.

As shown by the regression results in column (1), the coefficient of green credit is negative at the 10% significance level, indicating that green credit reduces the energy inputs required per unit of GDP and facilitates energy efficiency improvement. In column (2), the coefficient of green credit is positive at a significance level close to 10%, indicating green credit increases the proportion of clean energy in the energy consumption structure. Moreover, in columns (3) and (4), the coefficients of green credit are negative at the 5% significance level, indicating green credit reduces the proportions of coal-based energy and petroleum-based energy in the energy consumption structure. In summary, the empirical results in Table 3 show that, by increasing the proportion of clean energy and reducing the proportion of non-renewable energy, green credit can not only reduce total energy consumption and improve energy efficiency but also optimize the energy consumption structure, which consistent with the findings of Song et al. [33]. Since the implementation of the *2012 Green Credit Guidelines* has significantly reduced bank credit availability and credit allocation efficiency for energy-intensive enterprises [17], the sensible choice for affected enterprises is to proactively seek to transform and improve their energy consumption structure and energy efficiency performance to alleviate financial resource constraints. Consequently, green credit does promote green sustainable development at the upstream stage by optimizing energy inputs.

#### Technological innovation and green credit

Technological innovation is an important driving force for economic development and a practical path to solving environmental problems. This paper employs the quantity and proportion of green patents in each city to denote the degree of technological innovation that contributes to green and sustainable development. The criteria for recognition of green patents are based on the *IPC GREEN INVENTORY* issued by the World Intellectual Property Organization (WIPO). We obtain patent information from the State Intellectual Property Office Of The People’s Republic Of China based on Web Crawler and match the classification number of each patent with the *IPC GREEN INVENTORY* to identify green patents. Green patents are categorized into two groups: one is the green invention patent, which has outstanding substantive features and significant technical progress; the other is the green utility model patent, which has a relatively lower degree of inventiveness than the green invention patent. Considering the time lag of patent licensing, the quantity of patent applications is employed as the explained variable rather than patent grants.

The empirical results of the impact of green credit on green innovation are presented in Table 4. In Columns (1) to (3), the quantity of green patents is employed as explained variable, and the total quantity of patents is added to the model as a control variable to control for potential influencing factors involving innovation culture and R&D capacity at the city level. As seen in columns (1) and (2), the coefficients of green credit are significantly negative, and in column (3), the coefficient of green credit is negative but not that significant, which collectively imply green credit reduces green patents, especially for green invention patents with a high degree of inventiveness. Further, the percentage of green patents is used as explained variables in columns (4)-(6). The coefficients of green credit in columns (4) and (6) are insignificant, implying green credit has no significant effect on the proportion of green patents in total patents and the proportion of green utility model patents in green patents. But in column (5), green credit significantly reduces the proportion of green invention patents in green patents.

**Table 4:** Impact of green credit on green patents.

	(1)	(2)	(3)	(4)	(5)	(6)
	Quantity of green patents	Quantity of green invention patents	Quantity of green utility model patents	Proportion of green patents	Proportion of green invention patents	Proportion of green utility model patents
*gcredit*	-0.710**	-0.472**	-0.238	-0.746	-5.998**	0.306
	(-2.56)	(-2.31)	(-1.39)	(-1.25)	(-2.00)	(0.26)
Total quantity of patents	Yes	Yes	Yes	No	No	No
Control variable	Yes	Yes	Yes	Yes	Yes	Yes
Urban fixed effect	Yes	Yes	Yes	Yes	Yes	Yes
Year fixed effect	Yes	Yes	Yes	Yes	Yes	Yes
*N*	2151	2151	2151	2070	2154	2154
Adj.R^2^	0.743	0.607	0.719	0.110	0.092	0.044

Note:

***, **, and * mean significant at the 1%, 5%, and 10% levels, respectively; the values in parentheses are the t values adjusted by the clustering standard error; Adj.R^2^ represents adjusted R^2^.

The empirical results above indicate that at the current stage, green credit does not support green innovation and even crowds out green innovation with high technical value. These findings are potentially related to the following reasons: On the one hand, technological innovation is usually associated with high investment and high uncertainty, especially for green invention patents with significant technical progress. But for lenders, particularly banking institutions characterized by risk aversion, the eventual commercial purpose of credit business is the secure recovery of principal and interest. This potential conflict discourages banks from lending to green innovative enterprises and projects with a high degree of green inventiveness. Consequently, according to the data released by The People’s Bank of China, transportation, warehousing, and postal services have been the leading sectors in accessing green credit in recent years. However, the applicants for green patents in China are mainly energy enterprises and universities. On the other hand, for polluting and energy-intensive enterprises that are anxious to transform through technological innovation under various environmental regulations, the financial conditions to sustain research & development activities deteriorate further with the implementation of green credit policies. Besides, privately owned enterprises possess an average higher willingness and capability of green innovation than state-owned enterprises, but their accessibility to green credit is inferior due to ownership discrimination [47]. Our empirical findings that green credit negatively affects green innovation share similar viewpoints with Lu et al. [16], Luo et al. [48] and Wen et al. [17].

To ensure the results are not affected by other factors, the following robustness tests are conducted. First, all explained variables are replaced by the quantity and proportion of green patents granted, and the regression results are provided in S2 Table. Second, to take into account the innovation cycle, green credit variables are lagged by 1–4 periods and the regression results are provided in S3 Table. The empirical results are demonstrated to be robust.

#### Pollution outputs and green credit

Environmental conditions reflect the quality and sustainability of China’s economic development while affecting residents’ health, happiness, and security. The primary sources of environmental pollution, industrial soot emissions (*wsoot*), industrial wastewater discharge (*wwater*), and industrial nitrogen oxide emissions (*wno*), are selected to synthesize a comprehensive pollution emission index (*plt*) based on min-max normalization and Principal Component Analysis (PCA). The details are shown in Eqs (6)—(9).


$${plt}_{1jt}=\frac{{wsoot}_{jt}-\min\left({wsoot}_{jt}\right)}{\max\left({wsoot}_{jt}\right)-\min\left({wsoot}_{jt}\right)}$$ (6)

$${plt}_{2jt}=\frac{{wwater}_{jt}-min({wwater}_{jt})}{max({wwater}_{jt})-min({wwater}_{jt})}$$ (7)

$${plt}_{3jt}=\frac{{wno}_{jt}-min({wno}_{jt})}{max({wno}_{jt})-min({wno}_{jt})}$$ (8)

$${plt}_{jt}=\gamma_{1}{\times plt}_{1jt}+\gamma_{2}{\times plt}_{2jt}+\gamma_{3}{\times plt}_{3jt}$$ (9)

Column (1) in Table 5 provides the empirical results of pollution emissions and green credit. The coefficient of green credit is small in value and the t-test statistic is not significant, indicating the effect of green credit in reducing pollution emissions is not substantial. This finding may be due to the following reasons: First, banks have long preferred to grant credit to heavy industry sectors [49], which are the main industrial polluting sources in China. Even though green credit policies are implemented, it is challenging to reverse banks’ credit preferences in the short run, since most heavy industrial enterprises are state-owned and have assets as collateral which makes these customers ideal candidates for loan origination. Second, the financial constraints the green credit policies impose on highly leveraged enterprises intensify their greenwashing behavior [31]. Meanwhile, the inadequate professional competence in assessing environmental performance and the inconsistent standards and criteria for granting green credit across banks make it possible for enterprises to obtain credit through greenwashing behaviors. Third, the potential collusion between enterprises, local governments and even banks in the implementation of green credit policies prompted by each party’s performance pressure, may lead to a considerable gap between the actual and the desired policy effects [39]. Besides, other factors such as deficient environmental information disclosure, local protectionism, enterprise strategic polluting, and substitute financial resources also affect the effectiveness of green credit on pollution control.

**Table 5:** Impact of green credit on pollution reduction.

	(1)	(2)	(3)	(4)	(5)
	*plt*	*plt*	*plt*	*plt*	*plt*
*gcredit*	0.263	-1.139*	1.142**	-2.328***	0.816*
	(1.61)	(-1.69)	(2.38)	(-2.93)	(1.88)
Control variable	Yes	Yes	Yes	Yes	Yes
Urban fixed effect	Yes	Yes	Yes	Yes	Yes
Year fixed effect	Yes	Yes	Yes	Yes	Yes
*N*	2155	648	656	648	654
Adj.R^2^	0.474	0.204	0.270	0.131	0.205

Note:

***, **, and * mean significant at the 1%, 5%, and 10% levels, respectively; the values in parentheses are the t values adjusted by the clustering standard error; Adj.R^2^ represents adjusted R^2^. The explained variables are the comprehensive pollution index *plt*.

To ensure the robustness of the results, we average the various pollutants by weighting the regional GDP to synthesize a new comprehensive pollution emission index (rbplt). In addition, we also employ industrial soot emissions (plt1), industrial wastewater discharge (*plt*_*2*_) as well as industrial nitrogen oxide emissions (*plt*_*3*_) as the explained variables, respectively. The robustness test results in S4 Table demonstrate the robustness.

The heterogeneous binding effects of green credit on pollution reduction are further examined. First, cities are grouped by the ratio of interest expenditures of pollution-intensive industries to the interest expenditures of all manufacturing industries in the city. The 11 pollution-intensive industries are specified in *the First National Census of Pollution Sources* published by the State Council in 2006, including paper manufacturing, electricity, heat production, nonferrous metal smelting, etc. If a region has relatively high proportion of interest expenditures of polluting industries, it implies that the region is highly dependent on bank loans, which means it is more sensitive to bank credit policies. Such a region is defined as a region with strong green credit binding effects. In regions with strong green credit binding effects, enterprises are motivated to reduce pollution emissions to obtain bank loans. On the contrary, if the proportion of interest expenditures of the polluting industries in a region is low, the ability of bank credit to influence regional production and investment behaviors is relatively weak. Second, determine whether the polluting industries are the pillar industries in the region that have importance to local economic development and employment. Regions with polluting industries as ecnomic pillars are less constrained by green credit due to government intervention. Interest and asset data are from the National Bureau of Statistics of China.

Columns (2) and (3) of Table 5 are the results of grouped regression using the median proportion of interest expenditures of pollution-intensive industries in each year as the grouping variable. In Column (2), the interest expenditure of pollution-intensive industries is high and the coefficient of green credit is negative, indicating in regions with strong green credit binding effects, the pollution control effect is significant. On the contrary, column (3) shows the empirical results for the regions with low green credit binding effects. The coefficient of green credit is positive, which means green credit has negative influences on pollution control in these regions. It could be due to the strong financing capacity of pollution-intensive firms in these regions through various financing sources. Banks compete to extend credit to them, including green credit, to maintain business relationships. Columns (4) and (5) are the results of grouped regression using the median proportion of asset sizes of pollution-intensive industries in each year as the grouping variable. In Column (4), pollution-intensive industries are not pillar industries in these regions due to the low proportion of aggregated asset sizes of pollution-intensive industries in all manufacturing industries. In these regions, green credit effectively reduces pollution at a significance level of 1%, indicating green credit imposes strong binding effects in regions where pollution-intensive industries are not local economic pillars. On the contrary, in column (5), when the pollution-intensive industries are pillar industries in the regions, green credit has a negative influence on pollution reduction. It could be due to the implicit compromise and intervention of local governments for the stake of local economic development.

Accordingly, the results in Table 5 indicate green credit performs better pollution reduction effects in regions with relatively strong green credit binding effects and regions where pollution-intensive industries are not local economic pillars. The heterogeneity of pollution reduction effects of green credit has been explored to some degree in existing literature. For example, Zhang et al. [50] propose that the emission reduction effect of green credit is only evidenced in financially developed areas, with relatively more significant effects in resource-based areas. Our study complements the research findings on green credit’s heterogeneous influences on pollution reduction from the perspective of green credit binding effects and the importance of polluters to local economy.

### Robustness test

The robustness of the empirical results has been validated by changing the control variables of the basic model and by examining the heterogeneity of green credit effects over time, as discussed above. Furthermore, we continue by demonstrating that the results are less influenced by endogeneity issues. The primary sources of endogeneity usually involve two-way causality between the explanatory and dependent variables, missing variables, and measurement errors. Firstly, the weight in the calculation of the green credit index is the branch density of each bank in each region, which means the green credit variable is mainly affected by the geographical distribution of bank branches within the years studied. Theoretically, it is impractical for banks to decide how many branches they operate in a region base on the degree of the green sustainable development of this region. Thus, the problem of reverse causation should not be significant. Moreover, the results of the Granger causality test in S5 Table show that green credit is the Granger cause of GTFP, while GTFP is not the Granger cause of green credit. Therefore, the two-way causality between the explanatory and dependent variables should be limited. Secondly, the regional-level factors, including governmental factors (government intervention and environmental regulation), science and education factors (educational level and technology level), infrastructure factors (roads and internet), and financial factors, all have been well controlled in this paper. In addition, the confounding effect of regionally inherent factors on GTFP is effectively reduced by further controlling for region-level fixed effects. Consequently, the empirical results of this paper are less influenced by the omitted variables. Thirdly, to address the measurement error, green credit and GTFP are recalculated at the provincial level and the empirical results are shown in Table 6, which indicates a positive correlation still exists between green credit and GTFP.

**Table 6:** Examination of the relationship between green credit and GTFP at the provincial level.

	(1)
	*gtfp*
*gcredit*	0.309***
	(2.86)
Control variable	Yes
Urban fixed effect	Yes
Year fixed effect	Yes
*N*	238
Adj.R^2^	0.370

Note:

***, **, and * mean significant at the 1%, 5%, and 10% levels, respectively; the values in parentheses are the t values adjusted by the clustering standard error; Adj.R^2^ represents adjusted R^2^.

**Fig 1:**
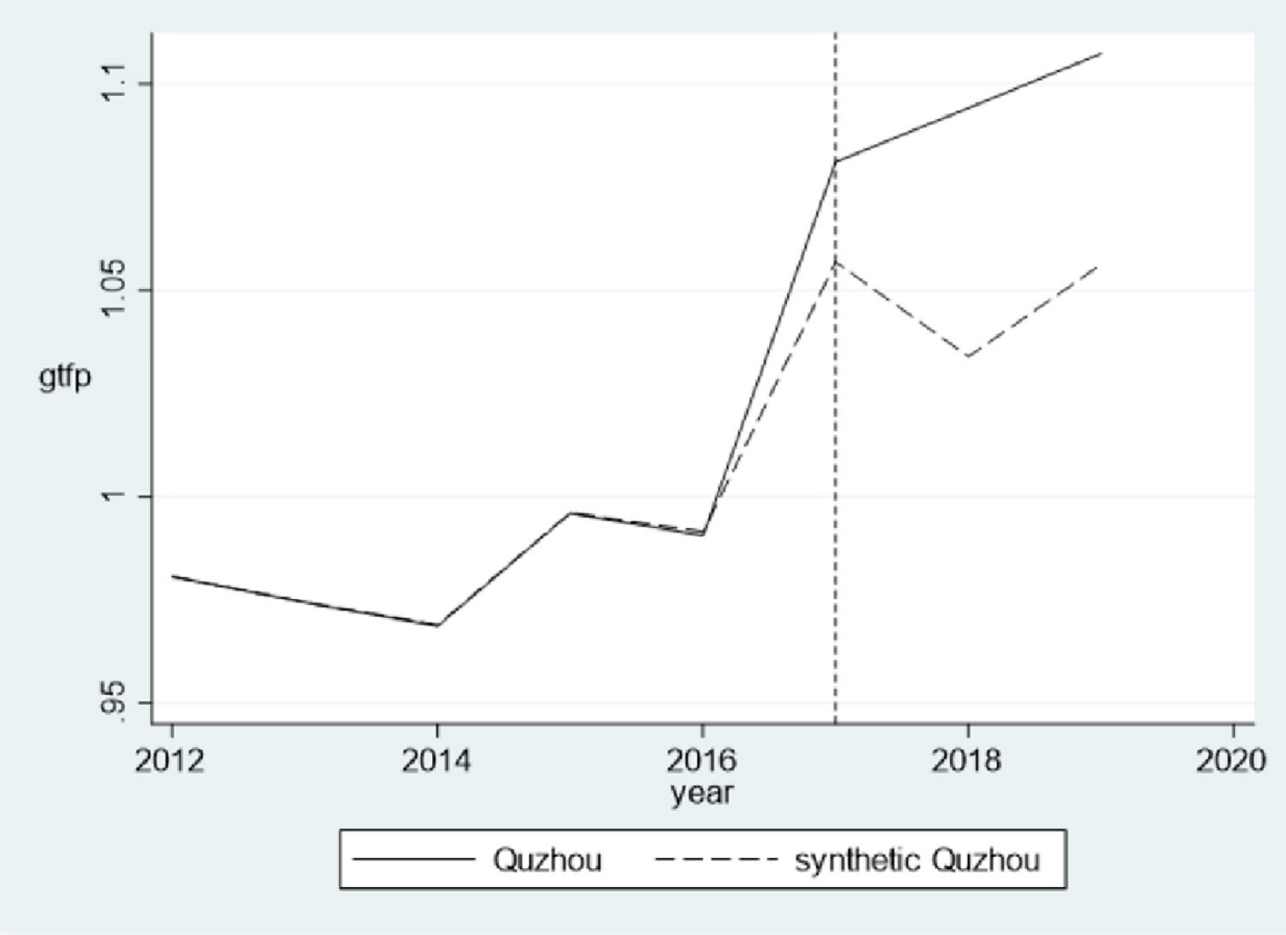
GTFP for real Quzhou and synthetic Quzhou.

In addition, the synthetic control method is applied to illustrate further that the empirical results are not affected by endogeneity. In June 2017, the State Council of China approved *the Overall Program Of Green Financial Reform And Innovation Pilot Zones*, and designated Quzhou, Huzhou, Guangzhou, Hami, Changji, Karamay, Guian, and Ganjiang as the pilot cities. Theoretically, the development of green credit in the pilot zones influenced by exogenous policies should increase significantly, which provides a valuable policy environment for identifying the impact of green credit. In policy evaluation, the counterfactual framework is often compared with the intervention results on actual data, and the difference between the two is the treatment effect. However, the difficulty often lies in how to observe counterfactual results, which are the outcomes that are not subject to policy intervention. The synthetic control method is employed to address this issue. The basic concept of the synthetic control method is to construct a fictitious synthetic control group by synthesizing other samples when the optimal control group cannot be found, and then compare the treatment group with the synthetic control group. Quzhou, one of the green financial reform and innovation pilot cities, is selected as the treatment group in our study. Sample cities for synthesizing the control group are selected from regions outside the provinces where the pilot cities locate. The details about the control group synthesizing and the related weights are shown in S6 Table, and the matching variables in actual Huzhou and synthetic Huzhou are shown in S7 Table. The year of the policy shock is set as 2017, consistent with the policy implementation year. The explained variable is GTFP, and the matching variables in the synthetic control framework include the control variables in the basic model and the GTFP from 2014 to 2016. Fig 1 and Table 7 present the results of the synthetic control method. Before 2017, the fit of GTFP between synthetic Quzhou and real Quzhou is very well. In 2017, Quzhou was designated as green financial reform and innovation pilot city, and since then GTFP of real Quzhou has increased significantly compared with synthetic Quzhou. This finding is consistent with the empirical results in this paper, which further validates the robustness of the positive relationship between green credit and GTFP.

**Table 7:** Results using the synthetic control method.

Year	Actual Quzhou	Synthetic Quzhou	Treatment Effect
2012	0.981	0.981	-0.000
2013	0.974	0.975	-0.000
2014	0.969	0.969	-0.000
2015	0.996	0.996	-0.000
2016	0.991	0.992	-0.001
2017	1.081	1.057	0.024
2018	1.094	1.034	0.060
2019	1.107	1.056	0.051

## Conclusions and policy implications

Green credit is not only the most significant component of green finance but also an vital force in promoting the overall green and low-carbon transformation of economic development in China. While green credit has gained noticeable growth in recent years, studies on the impact of green credit in China at the regional level are still insufficient. Many studies analyzed the impact of policy shocks based on the green credit policies implemented over ten years ago, but whether the findings are representative enough to explain the effects of green credit development in recent years is still pending further examined. This paper uses GTFP to quantify the degree of green sustainable development and examines the relationship between green sustainable development and green credit at the city level from 2012 to 2019. The empirical results indicate that green credit can promote green sustainable development and this positive effect gradually strengthens over time with the incremental implementations of complementary policies with substantial constraints and incentives. Along the value chain of influencing mechanism, in the upstream stage, green credit can reduce the total energy inputs and optimize the energy input structure. However, in the midstream stage, green credit cannot improve the level of green technological advancement and may even crowd out green inventions with high technical value. As for the downstream stage, green credit performs better pollution reduction effects in regions with relatively strong green credit binding effects or in regions where pollution-intensive industries are not local economic pillars.

Based on the conclusions above, the following policy recommendations are proposed to further reinforce the effectiveness of green credit:

First, enhance the green credit information disclosure mechanism and standardize the review, approval, and post-loan management processes of green credit, ensuring that green credit is genuinely invested in green projects. Currently, inadequate management and inefficient usage of green credit funds exist in the practice of green credit. The breadth, depth, and frequency of green credit information disclosure vary widely among enterprises. Efforts should be made to establish unified information disclosure standards, develop professional third-party green assessment agencies and build a shared green information platform. In particular, enterprises should take the initiative to undertake the primary responsibility of disclosing the specific use of green credit funds to effectively reduce information asymmetry between enterprises and financial institutions on green activities. While banks gain increasingly sufficient information on the environmental performance of enterprises, their environmental risk exposure would be reduced and their confidence in issuing green credit would be improved, which is conducive to the green transformation of enterprises and the sustainable development of the economy.

Second, with the endorsement of the government and regulators, promote the creation of diversified green credit products to facilitate their support for technological innovation and improve the return on investment. The characteristics of green innovation, such as significant investments, long cycles, and high uncertainty, conflict with the objectives of traditional credit products that focus on the safety of principal and stable returns. In the case of insufficient financial resources for green innovation due to market failure, it is more important for the government and regulators to guide and adjust relevant market activities actively. For example, government and regulators can improve the incentive mechanisms for green credit from the perspective of targeted requirement reserve ratio cuts, liquidity support from the central bank, risk-weighted capital adjustment, and tax benefits, to encourage a culture of product innovation and construct a diversified green credit products market. With government coordination, the positive externalities generated by green credit for environmental protection could be reasonably incorporated into banks’ return on investment. In this way, banks’ motivation to issue green credits to green innovation projects could be improved by gaining from the high return associated with the high risk of these projects.

Third, optimize banks’ green credit preferences and improve the economic and environmental benefits generated by green credit. Currently, green credit is more concentrated in transportation, warehousing, postal services, and energy sectors. In contrast, the supports for decarbonization projects and production upgrade projects in many pollution-intensive industries, such as steel, cement, and chemical sectors, are insufficient. Although these projects are launched by pollution-intensive enterprises, their environmental benefits could be significant. Therefore, as the green credit scale continuously expands, the matching between green capital supply and green development demand is pending improvement. Furthermore, a standardized environmental benefits evaluation system that covers energy conservation and environmental protection for green credit should be established to form a consistent interactive relationship between financial institutions and enterprises. The historical environmental benefits created by green credit should be used as a reference for the subsequent approval of green credit and the expected environmental benefits should be reflected in loan pricing or conditional rate to promote the goal achievement of green projects.

Although this paper provides valuable contributions to the research area of green credit and green sustainable development, specific limitations exist in the current study. Firstly, as the official information disclosure of green credit in China is not sufficient, existing studies, including ours, could only use estimation methods through different perspectives to measure green credit, which is overall logical and reasonable but may lose some accuracy. Secondly, since there might be a certain degree of lag in the effect of green credit on the economy, the dynamic impact and long-term effects of green credit deserve further study. Thirdly, considering the characteristics of the Chinese administrative systems, introducing the behavior of local governments and local officials into the analysis of green credit and green sustainable development could be valuable.

## Supporting information

S1 Table The units, data sources, measurements, and time periods of the variables. (DOCX)

S2 Table Robustness analysis of the relationship between green credit and green innovation. (DOCX)

S3 Table Robustness analysis of the relationship between green credit and green innovation with lagged explanatory variables. (DOCX)

S4 Table Robustness analysis of the relationship between green credit and environmental pollution. (DOCX)

S5 Table The results of Granger causality test. (DOCX)

S6 Table Cities in the control group and their weights for the synthetic control analysis. (DOCX)

S7 Table The matching variables in actual Huzhou and synthetic Huzhou. (DOCX)
