# Supplementary material for: Does green credit promote green sustainable development in regional economies?—Empirical evidence from 280 cities in China
Source: PLoS One. 2022 Nov 10;17(11):e0277569. doi: 10.1371/journal.pone.0277569 (PMC9648747; doi:10.1371/journal.pone.0277569)
Supplement: S1 Table — (DOCX) [file pone.0277569.s001.docx]

**S1 Table. The units, data sources, measurements, and time periods of the variables.**

| **Variable** | **unit** | **Sources** | **Measurement** | **Time period** |
| --- | --- | --- | --- | --- |
| **Energy consumption** | tce / ten million | China Energy Statistical Yearbook | energy consumption (tons of standard coal equivalent) / GDP | 2012—2019 |
| **Environmental pollutants** | 1 | China Statistical Yearbook on Environment | The details are shown in equations (6) - (9) | 2012—2019 |
| **GDP per capita** | yuan | CSMAR database | Ln(GDP/average annual population) | 2012—2019 |
| **Share of secondary industry in GDP** | 1 | CSMAR database | secondary industry GDP / GDP | 2012—2019 |
| **Share of tertiary industry in GDP** | 1 | CSMAR database | tertiary industry GDP / GDP | 2012—2019 |
| **Degree of financial development** | 1 | CSMAR database | RMB loan balance / GDP | 2012—2019 |
| **Degree of government intervention** | 1 | CSMAR database | (fiscal revenue - fiscal expenditure) / fiscal expenditure | 2012—2019 |
| **Education level** | 1 | China Statistical Yearbook | education spending / fiscal expenditure | 2012—2019 |
| **Technological level** | 1 | CSMAR database | technology spending / fiscal expenditure | 2012—2019 |
| **Openness level** | 1 | CSMAR database | actual foreign investment / GDP | 2012—2019 |
| **Domestic trade** | 1 | CSMAR database | total retail sales of consumer goods / GDP | 2012—2019 |
| **Urban road area per capita** | 1 | China Statistical Yearbook | urban road area per capita | 2012—2019 |
| **Degree of internet development** | 1 | China Statistical Yearbook | internet coverage | 2012—2019 |
| **stringency of local government environmental regulation** | 1 | manually collected city governments’ work reports | the ratio of ecological environment keywords to the total words of the governments’ work report | 2012—2019 |
| **Green patent** | 1 or % | China National Intellectual Property Administration | quantity and proportion of green patents | 2012—2019 |
