# Supplementary material for: Does green credit promote green sustainable development in regional economies?—Empirical evidence from 280 cities in China
Source: PLoS One. 2022 Nov 10;17(11):e0277569. doi: 10.1371/journal.pone.0277569 (PMC9648747; doi:10.1371/journal.pone.0277569)
Supplement: S2 Table — (DOCX) [file pone.0277569.s002.docx]

**S2 Table. Robustness analysis of the relationship between green credit and green innovation**

|  | **(1)** | **(2)** | **(3)** | **(4)** | **(5)** | **(6)** |
| --- | --- | --- | --- | --- | --- | --- |
|  | **Quantity of green patents** | **Quantity of green invention patents** | **Quantity of green utility model patents** | **Proportion of green patents** | **Proportion of green invention patents** | **Proportion of green utility model patents** |
| *gcredit* | -0.165 | -0.025 | -0.201 | 0.159 | -1.845 | -0.503 |
|  | (-0.54) | (-0.11) | (-1.06) | (0.36) | (-0.64) | (-0.45) |
| Control variable | Yes | Yes | Yes | Yes | Yes | Yes |
| Urban fixed effect | 2070 | 2071 | 2150 | 2070 | 2155 | 2155 |
| Year fixed effect | 0.721 | 0.441 | 0.705 | 0.226 | 0.022 | 0.065 |

Note: This table is basically the same as table 4, only the quantity and proportion of patent applications have been changed to the quantity and proportion of patents granted. The proportion of green patents in column (4) is measured by the proportion of the quantity of green patents in the total quantity of invention patents and utility model patents; the proportion of green inventions in column (5) is measured by the proportion of the quantity of green invention patents in the quantity of invention patents; and the proportion of green utility model patents is measured by the proportion of the quantity of green utility model patents in the quantity of utility model patents.
