# Supplementary material for: Does green credit promote green sustainable development in regional economies?—Empirical evidence from 280 cities in China
Source: PLoS One. 2022 Nov 10;17(11):e0277569. doi: 10.1371/journal.pone.0277569 (PMC9648747; doi:10.1371/journal.pone.0277569)
Supplement: S3 Table — (DOCX) [file pone.0277569.s003.docx]

**S3 Table. Robustness analysis of the relationship between green credit and green innovation with lagged explanatory variables**

|  | **(1)** | **(2)** | **(3)** | **(4)** | **(5)** | **(6)** |
| --- | --- | --- | --- | --- | --- | --- |
|  | **Number of green patents** | **Number of green inventions** | **Number of green utility models** | **Proportion of green patents** | **Proportion of green inventions** | **Proportion of green utility models** |
| *L.gcredit* | -0.679** | -0.195 | -0.484*** | -1.234 | -3.044* | -2.028 |
|  | (-1.99) | (-0.86) | (-2.95) | (-1.51) | (-1.96) | (-1.18) |
| *L2.gcredit* | -0.013 | -0.011 | -0.002 | -0.680* | -5.877*** | -0.273 |
|  | (-0.05) | (-0.06) | (-0.02) | (-1.71) | (-3.34) | (-0.26) |
| *L3.gcredit* | -0.323 | -0.210 | -0.113 | -2.230 | 1.730 | -5.969** |
|  | (-0.40) | (-0.30) | (-0.35) | (-1.50) | (0.23) | (-2.16) |
| *L4.gcredit* | -0.222 | 0.454 | -0.675 | 0.476 | 5.888 | -6.350 |
|  | (-0.18) | (0.48) | (-1.16) | (0.24) | (0.72) | (-1.55) |
| Control variable | Yes | Yes | Yes | Yes | Yes | Yes |
| Urban fixed effect | Yes | Yes | Yes | Yes | Yes | Yes |
| Year fixed effect | Yes | Yes | Yes | Yes | Yes | Yes |
| *N* | 967 | 967 | 967 | 952 | 968 | 968 |
| Adj.R^2^ | 0.815 | 0.382 | 0.886 | 0.230 | 0.103 | 0.068 |

Note: This table is basically the same as table 4; only the multiperiod lagged terms of the explanatory variables are included. L.gcredit is a one-period lagged green credit variable, L2.gcredit is a two-period lagged green credit variable, and so on.
