# Supplementary material for: Does green credit promote green sustainable development in regional economies?—Empirical evidence from 280 cities in China
Source: PLoS One. 2022 Nov 10;17(11):e0277569. doi: 10.1371/journal.pone.0277569 (PMC9648747; doi:10.1371/journal.pone.0277569)
Supplement: S4 Table — (DOCX) [file pone.0277569.s004.docx]

**S4 Table. Robustness analysis of the relationship between green credit and environmental pollution**

|  | **(1)** | **(2)** | **(3)** | **(4)** |
| --- | --- | --- | --- | --- |
|  | ***rbplt*** | ***plt_1_*** | ***plt_2_*** | ***plt_3_*** |
| *gcredit* | 6.471 | 0.003 | -0.072 | 11.748** |
|  | (1.03) | (0.08) | (-0.51) | (2.09) |
| Control variable | Yes | Yes | Yes | Yes |
| Urban fixed effect | Yes | Yes | Yes | Yes |
| Year fixed effect | Yes | Yes | Yes | Yes |
| *N* | 2150 | 2155 | 2155 | 2155 |
| Adj.R^2^ | 0.003 | 0.180 | 0.021 | 0.016 |

Note: The explained variable in column (1) is the comprehensive pollutant emission index weighted by regional GDP (*rbplt*), and the explained variables in columns (2)-(4) are industrial soot emissions (*plt1*), industrial wastewater discharge (*plt2*), and industrial nitrogen oxide emissions (*plt3*), respectively.
