# Supplementary material for: Does green credit promote green sustainable development in regional economies?—Empirical evidence from 280 cities in China
Source: PLoS One. 2022 Nov 10;17(11):e0277569. doi: 10.1371/journal.pone.0277569 (PMC9648747; doi:10.1371/journal.pone.0277569)
Supplement: S5 Table — (DOCX) [file pone.0277569.s005.docx]

**S5 Table. The results of Granger causality test**

|  | **(1)** | **(2)** |
| --- | --- | --- |
|  | gtfp | gcredit |
| L.gcredit | 0.661^***^ |  |
|  | (18.28) |  |
| L2.gcredit | 0.839^***^ |  |
|  | (21.45) |  |
| L.gtfp |  | 0.056^*^ |
|  |  | (1.68) |
| L2.gtfp |  | 0.055 |
|  |  | (1.62) |
| *N* | 218 | 218 |
| Wald_HPJ | 1022.811 | 4.322 |
| P-value | 0.000 | 0.115 |
