# Supplementary material for: Does green credit promote green sustainable development in regional economies?—Empirical evidence from 280 cities in China
Source: PLoS One. 2022 Nov 10;17(11):e0277569. doi: 10.1371/journal.pone.0277569 (PMC9648747; doi:10.1371/journal.pone.0277569)
Supplement: S6 Table — (DOCX) [file pone.0277569.s006.docx]

**S6 Table. Cities in the control group and their weights for the synthetic control analysis**

| **City in Control group** | **Weight** |
| --- | --- |
| Sanming | 0.171 |
| Lishui | 0.182 |
| Shiyan | 0.174 |
| Lu Liang | 0.035 |
| Panzhi | 0.004 |
| Zibo | 0.059 |
| Jingzhou | 0.154 |
| Heze | 0.047 |
| Jinhua | 0.174 |
