# Supplementary material for: Does green credit promote green sustainable development in regional economies?—Empirical evidence from 280 cities in China
Source: PLoS One. 2022 Nov 10;17(11):e0277569. doi: 10.1371/journal.pone.0277569 (PMC9648747; doi:10.1371/journal.pone.0277569)
Supplement: S7 Table — (DOCX) [file pone.0277569.s007.docx]

**S7 Table. The** **matching variables in actual Huzhou and synthetic Huzhou**

| **Matching variable** | **Actual Huzhou** | **Synthetic Huzhou** |
| --- | --- | --- |
| Percentage of the secondary industry in GDP | 49.524 | 49.448 |
| Percentage of the tertiary industry in GDP | 42.898 | 38.914 |
| GDP per capita | 10.677 | 10.640 |
| Level of financial development | 1.254 | 1.014 |
| Education level | 0.193 | 0.194 |
| Level of financial development | 1.254 | 1.014 |
| Openness level | 0.449 | 0.448 |
| Science and technology level | 0.029 | 0.020 |
| Degree of internet development | 0.189 | 0.208 |
| Domestic trade | 0.001 | 0.001 |
| Environmental regulation | 0.009 | 0.008 |
| Urban road area per capita | 17.992 | 17.256 |
| GTFP 2014 | 0.969 | 0.969 |
| GTFP 2015 | 0.996 | 0.996 |
| GTFP 2016 | 0.991 | 0.992 |
